# KRAS G12D targeted therapies for pancreatic cancer: Has the fortress been conquered?

**DOI:** 10.3389/fonc.2022.1013902

**Published:** 2022-11-29

**Authors:** Sahar F. Bannoura, Husain Yar Khan, Asfar S. Azmi

**Affiliations:** ^1^ Cancer Biology Graduate Program, Wayne State University School of Medicine, Karmanos Cancer Institute, Detroit, MI, United States; ^2^ Department of Oncology, Karmanos Cancer Institute, Wayne State University School of Medicine, Detroit, MI, United States

**Keywords:** KRAS, KRAS G12C, KRAS G12D, sotorasib, adagrasib, MRTX1133, adoptive cell therapy, immunotherapy

## Abstract

KRAS mutations are among the most commonly occurring mutations in cancer. After being deemed undruggable for decades, KRAS G12C specific inhibitors showed that small molecule inhibitors can be developed against this notorious target. At the same time, there is still no agent that could target KRAS G12D which is the most common KRAS mutation and is found in the majority of KRAS-mutated pancreatic tumors. Nevertheless, significant progress is now being made in the G12D space with the development of several compounds that can bind to and inhibit KRAS G12D, most notably MRTX1133. Exciting advances in this field also include an immunotherapeutic approach that uses adoptive T-cell transfer to specifically target G12D in pancreatic cancer. In this mini-review, we discuss recent advances in KRAS G12D targeting and the potential for further clinical development of the various approaches.

## Introduction

RAS genes (KRAS, HRAS, NRAS) are the most commonly mutated oncogenes in cancers, resulting in increased downstream signaling that drives incessant proliferation and tumorigenesis. Certain tumors are more dependent on KRAS mutation, especially pancreatic ductal adenocarcinoma (PDAC) which remains one of the most lethal cancers, with a 5-year survival rate of 11% in the United States (1). Advances in our understanding of PDAC molecular pathology and subtypes have not translated into significant improvements in patient outcomes (2). Genetic alterations in the oncogene KRAS, and the tumor suppressors CDKN2A, SMAD4, and TP53 are the most common mutational drivers in PDAC, in addition to various genes identified at low mutation frequency. This complex genetic landscape has created a tremendous challenge for therapeutic targeting.

## KRAS mutations in PDAC

**Figure 1:**
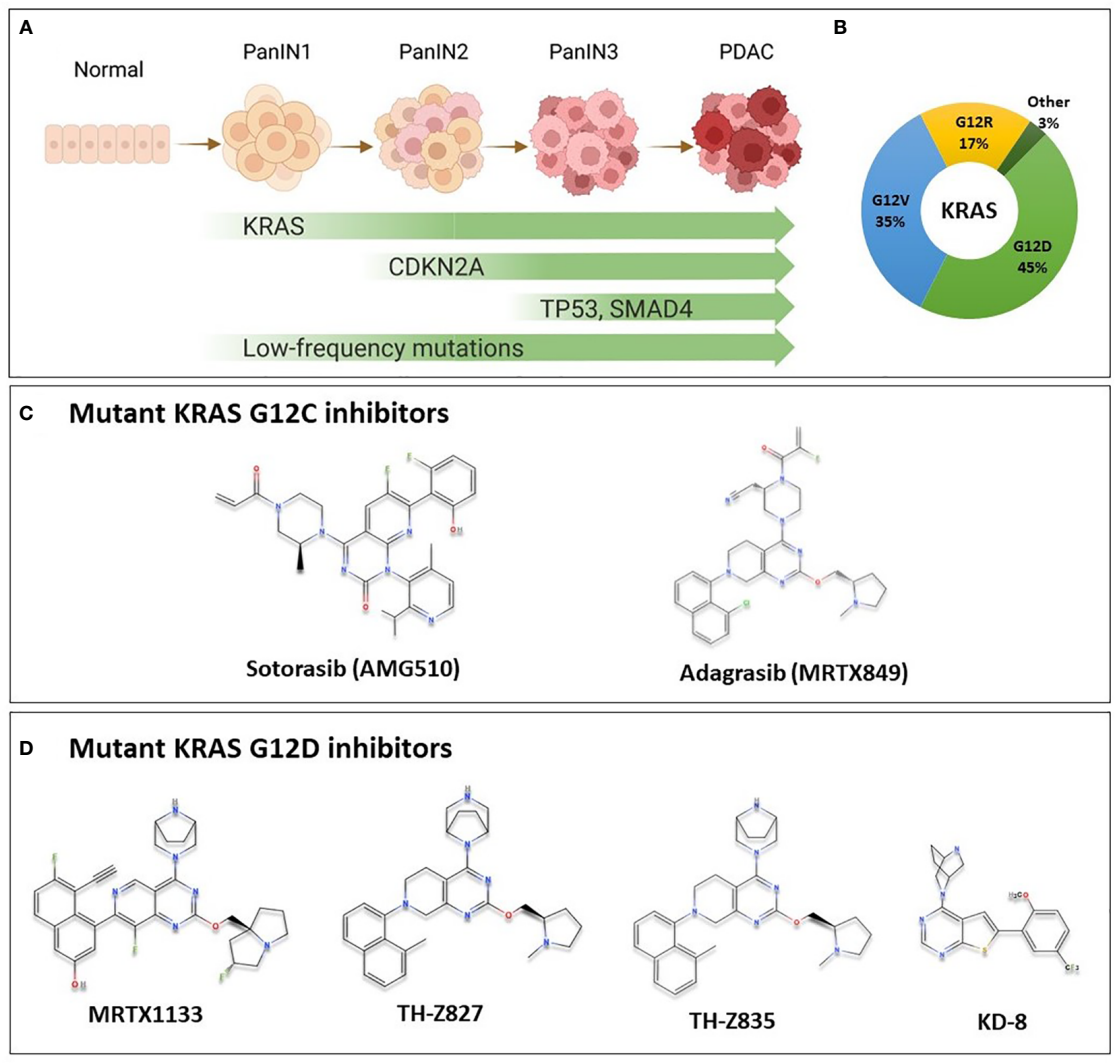
KRAS Oncogene in PDAC and the chemical structure of some of mutant KRAS small molecule inhibitors. **(A)** Multi-stage development model of KRAS-mutated PDAC. As cells acquire mutations in KRAS, CDKN2A, SMAD4 and TP53 in addition to less commonly mutated genes, the lesion progresses from low grade pancreatic intraepithelial neoplasia (PanIN1), through PanIN2, and high grade PanIN3 to become invasive carcinoma. **(B)** Most common KRAS mutations in PDAC are G12D (45%), G12V (35%), G12R (17%) representing important targets for PDAC patients. Other mutations such as G12C, G12A and others are less common (3%). **(C)** Sotorasib and adagrasib are mutant KRAS G12C inhibitors. They bind specifically to the mutant form by making covalent interactions with Cys12 in the switch-II pocket of mutant KRAS, locking it in the inactive state, and preventing downstream signaling. These agents have been investigated in clinical trials, and sotorasib is currently FDA approved for the treatment of KRAS G12C-mutated NSCLC. **(D)** Novel KRAS G12D inhibitors with available chemical structures, MRTX1133, TH-Z827, TH-Z835, and KD-8. These agents bind specifically and non-covalently to mutant KRAS G12D, thereby inhibiting proliferation in mutated cells. These agents are currently in preclinical development, and details on further clinical development are not currently known.

Mutations in KRAS, CDKN2A, TP53, and SMAD4 are the major genetic mutations that underly PDAC development (**Figure 1A**). The KRAS oncogene is the major oncogenic driver of PDAC (86 – 91%) (3), and is considered a master oncogenic regulator that drives cancer hallmarks including sustained proliferative signaling and evading growth suppression. KRAS mutations are found at the earliest stage of PDAC development in patient samples, and are required for the initiation and maintenance of PDAC in genetically engineered mouse models, suggesting that KRAS mutations are important for both PDAC initiation and progression (4–6). The most predominant KRAS mutation site in PDAC occurs at codon 12; most commonly G12D (45%), followed by G12V (35%), and G12R at 17% (**Figure 1B**) (3). Other mutations such as G12C and G12F occur at a lower frequency.

Mutant KRAS is notoriously difficult to target. It had been deemed undruggable until the recent success of inhibitors that target KRAS with a G12C mutation, AMG510 (sotorasib) and MRTX849 (adagrasib) (**Figure 1C**) (7). Sotorasib has been granted approval by the US Food and Drug Administration (FDA) for the treatment of certain patients with KRAS-mutated non-small cell lung cancer (NSCLC); and adagrasib has been given a breakthrough therapy designation by the FDA also for NSCLC (8). For PDAC patients, however, a mutation that replaces Glycine at codon 12 with Aspartic Acid (G12D) is the most common mutational event in KRAS, representing approximately 45% of KRAS mutations, as previously mentioned (3), and only a very modest proportion of patients would benefit from G12C targeted therapy. Nonetheless, since KRAS was found to be potentially druggable, multiple groups have aptly been working to develop inhibitors targeting the most common KRAS G12D mutation.

## Overview of KRAS oncogene and targeting efforts

KRAS is a small GTPase protein that is encoded by the *KRAS* proto-oncogene (9). It is activated by extracellular growth signaling that is transmitted by growth receptors such as EGFR family (10). Growth signaling allows KRAS to exchange its GDP for GTP, thereby affecting conformational change and further transmission of downstream signals. KRAS is upstream of critical signaling pathways, such as RAF/MEK/ERK MAPK, and PI3K signaling pathways.

KRAS cycles between GDP and GTP with the activity of guanine exchange factors (GEFs) that catalyze the exchange of GDP for GTP, and GTPase activating proteins (GAPs) that enhance the intrinsic KRAS GTPase activity. Son of Sevenless 1 (SOS1) is the main GEF to act on KRAS, and together with GAPs, it is responsible for the regulation of the guanine exchange cycle, and maintenance of KRAS in an inactive state in the absence of proper growth signaling (11). Mutations in KRAS change the dynamics of the guanine exchange cycle, resulting in hyperactive KRAS and increased pools of GTP-bound KRAS. This leads to the constant activation of downstream signaling cascades such as MAPK and PI3K, and consequently incessant cellular proliferation.

KRAS is frequently mutated in pancreatic cancer, colorectal cancer (CRC), and lung cancer (3). Therefore, looking for ways to target KRAS, whether directly or indirectly, has garnered great attention from researchers (12). Directly targeting KRAS had been elusive for decades. KRAS lacks a suitable deep hydrophobic binding pocket to design small molecule inhibitors, other than the GTP/GDP binding pocket. However, GTP is highly abundant in the cell, thus precluding efficacious nucleotide competitive inhibitors. Additionally, indirect targeting of KRAS through upstream or downstream regulators or effectors was either ineffective or caused major toxicities (13). The major breakthrough in the field came when undruggable KRAS was finally drugged with the discovery of the first KRAS G12C inhibitors.

## The Discovery of KRAS G12C inhibitors and its targetable pocket

Pioneering work by Shokat and colleagues paved the way for the clinical development of mutant KRAS G12C inhibitors (14). They were able to identify a druggable pocket termed the switch II pocket, which contains a reactive cysteine as a result of the G12C mutation in mutant KRAS (14). The co-crystal structure of hit compound and KRAS showed the compound binding in a pocket that lies beneath the switch II region of KRAS protein (14). Within the switch II allosteric pocket Cys12 is present, which contains a nucleophilic thiol group, making it a prime target for covalent drugs (15). It also affords the inhibitor high specificity over the other RAS isoforms (HRAS and NRAS) as well as wild-type KRAS, thereby reducing off-target effects and toxicities.

The early G12C inhibitor, compound 12, is a covalent inhibitor that binds in the switch II pocket of KRAS G12C in its GDP-bound form. Several other compounds were subsequently discovered and reported to have activity against cancer cells with KRAS G12C mutations (16, 17). An early compound, ARS-1620, was shown to inhibit tumor growth in cell-derived and patient-derived xenograft models (18). These efforts provided proof of concept for targeting KRAS G12C, which culminated in the development of the clinical agents sotorasib and adagrasib (19–23). Based on the safety, tolerability, and efficacy results of a phase 1/2 study of sotorasib (CodeBreaK 100, NCT03600883), it has received FDA approval for advanced NSCLC with KRAS G12C mutations. Results from the trial demonstrated an 80.6% disease control rate, with four patients achieving a complete response, and a median progression free survival of 6.3 months (22). A phase III trial (CodeBreaK 200, NCT04303780) is ongoing, which compares sotorasib with docetaxel chemotherapy in previously treated NSCLC patients. Sotorasib is also being investigated clinically as first-line therapy for stage 4 NSCLC patients (NCT04933695).

Adagrasib is being investigated in a phase I/II clinical trial (KRYSTAL-1, NCT03785249). In patients with measurable disease at baseline, the objective response rate was 42.9%, median progression free survival of 6.5 months, and overall survival of 12.6 months after 15.6 months of follow-up (23).

The limitation of any single agent treatment is the inevitable emergence of resistance. Studies have shown that resistance to G12C inhibitors could be inherent or acquired (24). Mechanisms of resistance include activation of receptor tyrosine kinase (RTKs), resulting in downstream activation of KRAS *via* SHP2 (25). Several mechanisms of resistance have been reported in patient samples treated with adagrasib including secondary activating mutations in KRAS, amplification of KRAS(G12C), and mutations in compensatory pathways that bypass KRAS such as MET amplification, and NRAS, BRAF, MAP2K1, and RET mutations (26). To combat resistance to G12C inhibitors, several trials are looking to combine mutation specific inhibitors with other agents. In CodeBreaK 101 (NCT04185883) several drugs are being investigated in combination with sotorasib, such as trametinib, TNO155, everolimus, palbociclib, pembrolizumab or several other agents. Adagrasib is also being evaluated in combination with other agents, such as pembrolizumab (NCT04613596), and TNO155 (NCT04330664). Although these trials would primarily benefit lung cancer patients, they provide valuable strategies for future targeting of non-G12C mutations.

As discussed, sotorasib has been given FDA approval for the treatment of certain patients with NSCLC (27). Despite encouraging results from the trials, and FDA approval, these drugs have provided little hope for the majority of PDAC patients. While KRAS G12C mutations are more prevalent in lung cancer, KRAS G12D mutation is the most common in PDAC as well as CRC. Available G12C inhibitors rely on the reactivity of the thiol group in Cys12 of mutant KRAS. To benefit PDAC patients and target the G12D mutation, an approach that does not rely on a reactive cysteine is required.

## Small molecule inhibitors targeting KRAS G12D

### Discovery of KRAS G12D inhibitor MRTX1133

As previously mentioned, G12D mutations are present in a large proportion of PDAC patients. The covalent G12C inhibitors relied on the presence of a strongly nucleophilic cysteine in the switch II pocket, as well as the unique biochemical properties of the KRAS G12C mutant, which has a higher rate of GTP hydrolysis compared to other mutants (28). The switch II pocket in KRAS G12D, however, lacks a reactive cysteine to target with a covalent inhibitor, and aspartic acid is not considered a good target for covalent attack.

A structure-based medicinal chemistry approach has been employed to identify compounds that can react through a salt bridge with Asp12 of the switch II pocket in KRAS G12D (29). Using adagrasib as a starting point, chemical modifications to the reactive warhead and various other groups in the compound were introduced to increase the binding affinity within the pocket. Optimization of hit compounds resulted in the discovery of MRTX1133 (**Figure 1D**), which selectively and reversibly binds KRAS G12D with low nanomolar affinity in cellular assays (30).

Binding of MRTX1133 to KRAS G12D prevents downstream signaling through inhibition of nucleotide exchange and binding of downstream effector RAF1 (30). This compound was shown to inhibit oncogenic KRAS signaling selectively in tumor cells (30). In a xenograft mouse model, MRTX1133 was able to significantly reduce tumor growth and decrease the phosphorylation of downstream signaling molecule ERK in a dose-dependent manner (30). So far, this agent remains an *in vitro* tool compound, and its clinical progress is not known.

### Other KRAS G12D inhibitors

Besides MRTX1133 described above, other groups have been pursuing the development of KRAS G12D inhibitors as well. For example, using a medicinal chemistry approach to find inhibitors that bind to Asp12 in KRAS G12D, two inhibitors TH-Z827 and TH-Z835 were discovered (**Figure 1D**) (31). These inhibitors form a salt bridge with Asp12 within the switch-II pocket resulting in the inhibition of KRAS signaling in G12D mutant PDAC cell lines (31). These compounds were able to bind the G12D mutant specifically, and not KRAS G12C or WT. The compounds also showed *in vivo* inhibition of tumor growth in xenograft tumor mouse models (31). Another set of inhibitors was discovered using virtual combinatorial chemistry and compound screening approach (32). In this approach, they used the backbone of G12C inhibitors in combination with piperazine-based compounds as building blocks for the compound library, followed by molecular docking to discover compounds with predicted selective binding. Compound ‘KD-8’ was discovered, which resulted in the inhibition of cellular and tumor growth of KRAS G12D mutated cells (32). However, further development of these compounds is required to increase their potency and minimize off-target effects. While these agents are not yet ready for clinical development, they provide further proof of principle that several classes of G12D inhibitors could be potentially discovered as clinical anti-cancer agents.

A tricomplex inhibitor, RMC-9805, is a novel covalent KRAS G12D inhibitor that binds KRAS in the GTP-bound state, thus termed a KRAS-G12D(ON) inhibitor (33). Tricomplex inhibitors bind a chaperone protein, Cyclophilin A, which is ubiquitously found inside the cell (34), which then binds the target protein, creating a target-inhibitor-Cyclophilin-A complex. RMC-9805 reacts covalently with Asp12, thereby attenuating KRAS G12D downstream signaling specifically over KRAS WT and other KRAS mutants, and it restricts tumor growth in xenograft PDAC and CRC mouse models (33). Within this class of inhibitors, there are several other compounds being developed to target various KRAS mutants, as well.

An emerging strategy to target oncogenic RAS is using monobodies to target the α4-α5 interface of KRAS, thus preventing its dimerization and downstream signaling (35–37). A monobody termed NS-1 was able to inhibit growth of G12D mutated PDAC in mice (35). However, the limitation of this strategy is the low cellular permeability of these large molecules. Therefore, further optimization is required for this approach prior to clinical testing.

Various efforts are now focused on research and discovery of novel KRAS G12D inhibitors. The development of agents, such as MRTX1133, is exciting for the field of PDAC research and provides evidence that KRAS G12D can be effectively targeted with a small molecule inhibitor. As with the G12C inhibitors, perhaps this discovery could lead to more small molecules entering the arsenal against G12D. As we await to see further clinical development of MRTX1133, the optimization of hits and potential discovery of more targeted compounds is anticipated. These efforts are critical for patients with KRAS G12D mutations, which make up a large subset of Ras-mutated cancers, especially for PDAC where the therapeutics currently available are providing meager benefits in the majority of cases.

## Immunotherapy targeting KRAS G12D in PDAC

Immunotherapeutic approaches have not had great success for PDAC patients (38). In other Ras-mutated cancers, immune checkpoint inhibitors (ICIs) have improved patient outcomes in clinical trials and thus have been approved by the FDA for the treatment of NSCLC and melanoma (39). However, ICIs did not achieve similar success in PDAC, even in combinatorial approaches. PDAC is characterized by a desmoplastic tumor microenvironment (TME) with low numbers of tumor-infiltrating lymphocytes (TILs) and is largely considered to be immunosuppressive (40). Immune cells that are found within the PDAC TME include tumor-associated macrophages (TAMs), myeloid-derived suppressive cells (MDSCs), and regulatory T cells (Tregs) which contribute to immune evasion, PDAC progression, and resistance to immunotherapies (38, 41). These factors could explain the observed low response of PDAC to ICIs in clinical trials (42–44). The exception is a small subset of PDAC patients with high microsatellite instability (MSI-H) tumors or mismatch repair deficiency (<1%) who can benefit from anti-PD-1 therapy (45, 46). Nevertheless, attempts are being made to identify targets that could reinvigorate the immune suppressive tumor microenvironment to become more immunogenic and better responsive to immunotherapy.

### Mutant KRAS modulates tumor immune microenvironment

Several studies have highlighted the role of oncogenic KRAS in establishing a pro-inflammatory microenvironment that enables PDAC tumorigenesis and progression (47–50). Oncogenic KRAS G12D signaling in tumor cells regulates the signaling of surrounding stromal cells and establishes reciprocal signaling between tumor and stromal cells (49, 51). PD-L1 expression is also suppressed by oncogenic KRAS G12D, and patients with G12D mutation have lower PD-L1 expression compared to other KRAS mutants (52, 53). Additional evidence for the role of oncogenic KRAS in modulating the TME comes from a recent study utilizing the anti-KRAS G12C agent AMG510 (19); where treatment with AMG510 increased the number of cytotoxic CD8+ T cells that infiltrated tumors in mice. Anti-KRAS therapy also synergized with anti-PD-1 treatment and produced durable anti-tumor responses (19). This provides strong evidence that KRAS signaling is involved in modulating the TME and maintaining an immune evasive environment, which hinders the development of ICIs as monotherapy in PDAC. Nevertheless, it also shows that using the right tools, the TME can be modulated and PDAC can potentially become responsive to immunotherapeutic approaches.

### T cell therapy targeting KRAS G12D

Adoptive cell therapy utilizes the patient’s own lymphocytes which may be engineered to express receptors that specifically target tumor neoantigens (54). This immunotherapeutic approach may be suitable to target KRAS neoantigens for PDAC therapy and to address the challenge of targeting KRAS, as well as the challenge of implementation of immunotherapies in PDAC. A study identified HLA-A*11:01 to be able to present KRAS neoantigens, and then generated murine T cells that recognize G12D mutated PDAC in an HLA-A*11:01 restricted manner and could inhibit the growth of tumors *in vivo*. (55). An ongoing phase I/II clinical trial is investigating transfer of T-cells engineered to express a G12D specific murine T-cell receptor (TCR) in HLA-A*11:01 patients with solid tumors, including pancreatic cancer, harboring the KRAS G12D mutation (NCT03745326).

**Figure 2:**
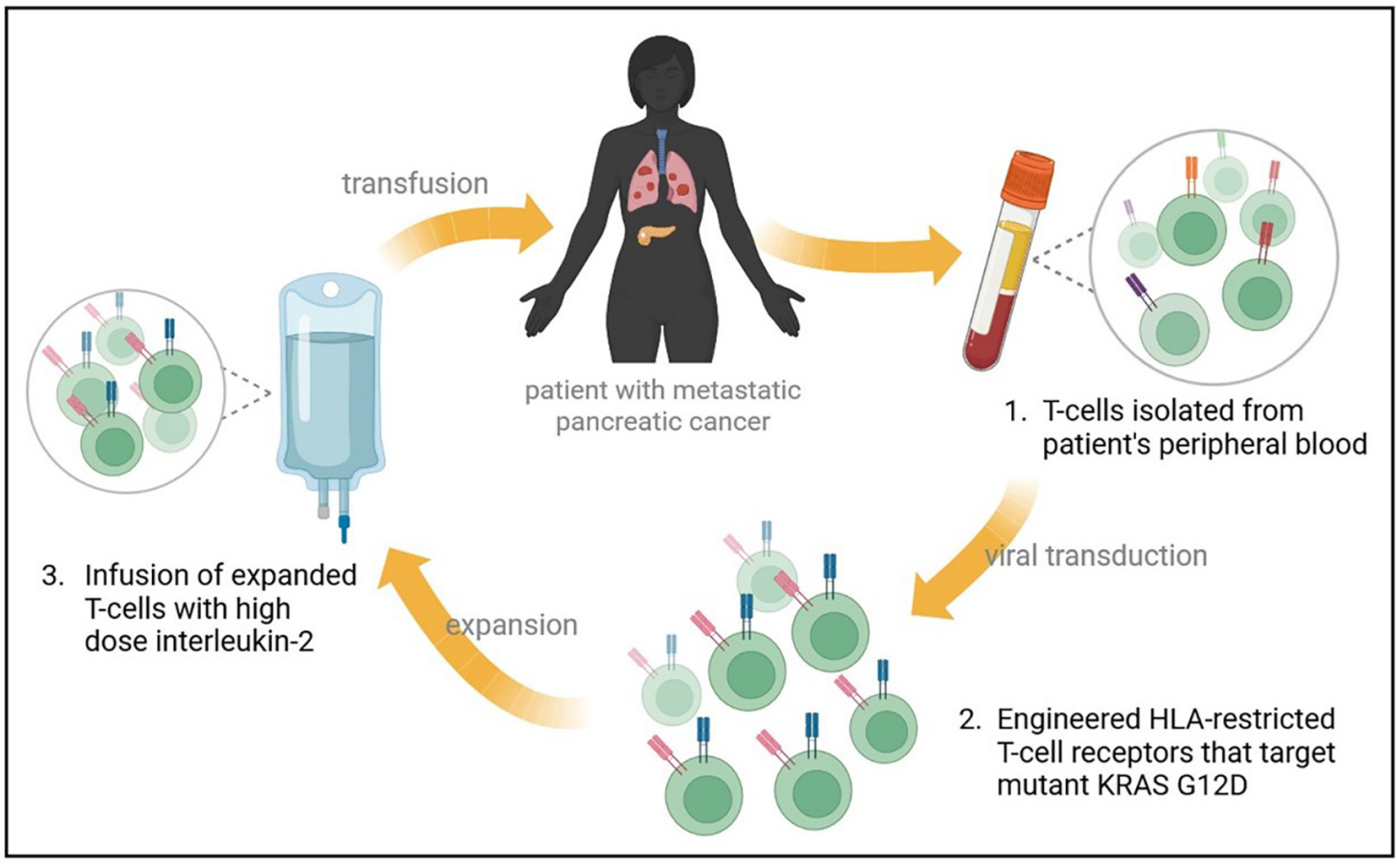
Treatment of metastatic PDAC with mutant KRAS-targeting genetically engineered T-cells. A heavily pretreated patient with metastatic PDAC to the lungs underwent an expiremental immunotherapy that targets mutant KRAS. Autologous T-cells were isolated and transfected with genes encoding two HLA-restricted T-cell receptors targeting KRAS G12D epitopes. The infusion product contained 16.2x10^9^ T cells, and supportive high dose interleukin-2 therapy was administered. Metastatic lung lesions regressed at 1 month follow up, with continued regression at 6-months and an overall partial response rate of 72%.

In a recent report, a patient with metastatic G12D-mutated PDAC received adoptive cell transfer therapy using engineered autologous T cells that target KRAS G12D mutant protein in the tumor, resulting in regression of metastases in the patient (**Figure 2**) (56). A patient with CRC had been previously reported to receive an infusion of ex-vivo expanded T cells with HLA-C*08:02 restriction and KRAS G12D reactivity, resulting in the regression of metastatic lung lesions (57). Based on the previous success of this approach, a heavily pretreated PDAC patient with lung metastases was treated with T-cells that were engineered to express HLA-C*08:02 restricted TCRs with specificity against KRAS G12D (56). A single infusion of 16.2×10^9^ T-cells was given to the patient, which contained 85% CD8+ T-cells, and 15% CD4+ T cells, with high-dose interleukin-2 therapy to support T-cell expansion. One month follow-up revealed regression of metastatic lung lesions, which continued to regress at the 6-month follow-up with an overall objective partial response of 72% according to RECIST, version 1.1, criteria (56). Additionally, the infused T-cells were found to persist in circulation at 6 months, making up 2.4% of total circulating T cells.

This reported case shows that engineered TCRs could be beneficial to some patients with metastatic PDAC. The drawback of this therapy is that TCRs were restricted by a specific HLA-C*08:02, which is expressed by a small subset of patients. Further studies and clinical trials are needed to investigate this therapy on a larger scale, and to identify other KRAS-G12D-reactive TCRs which could be utilized for similar therapies (58, 59). Meanwhile, results from the ongoing clinical trial for HLA-A*11:01+ patients are eagerly awaited.

## Targeting KRAS mutations beyond G12D

Since the discovery of G12C inhibitors, there has been growing interest in finding novel mutant-specific inhibitors to other common KRAS mutants. For a more detailed review, Nagasaka and colleagues have recently published an article highlighting novel strategies to target KRAS beyond G12C inhibitors including cancer vaccines, adoptive cell therapy, PROTACs and CRISPR/Cas9 (60).

KRAS G12R mutation is present in 17% of PDAC cases (**Figure 1**) and is therefore an important target for PDAC. Recent work has shown that the mutant arginine 12 in KRAS G12R can be targeted covalently with small molecule electrophiles (61). Although the molecules that target G12R have not shown activity in mutant cells, they could be further optimized to develop more potent molecules, and thus this presents an important proof of concept for targeting G12R. Less prevalent in PDAC, the G12S mutation in KRAS has also been targeted recently with compounds that acylate the noncatalytic mutant serine 12 residue (62). These compounds have shown selectivity for cells harboring the G12S mutation, but currently remain *in vitro* tools that require further optimization. Another class of drugs mentioned previously, is the tricomplex Ras(On) inhibitors, which are being developed against various KRAS mutants including G12C, G12D, and G13C, in addition to a G12X inhibitor that targets several G12 mutants (63).

## Conclusion

RAS genes are the most commonly mutated oncogenes in cancers. A mutation in KRAS is the most common oncogenic driver in PDAC and is also a known driver of NSCLC and CRC. Until the recent discovery of KRAS G12C inhibitors, KRAS had been considered an undruggable target for decades (64). Given the success of translating this discovery to the clinic, research efforts are focusing on drugging KRAS G12D, the most common hotspot mutation. The discovery of the preclinical agent MRTX1133 is an exciting advance for pancreatic cancer research. MRTX1133 binds potently and reversibly in the switch II pocket of mutant KRAS G12D. We hope that this discovery will lead to precipitous efforts by various groups to introduce optimized agents that can be tested clinically for PDAC patients.

The switch-II pocket seems to be a druggable pocket in various KRAS mutants and is susceptible to reversible non-covalent inhibition. Additionally, this pocket could potentially be targeted in both the GDP as well as the GTP-bound states of KRAS (31, 65), meaning it is possible to develop inhibitors that target KRAS mutants with low intrinsic GTPase activity (28).

Cancer immunotherapy using ICIs and the observed durable responses with limited toxicities has generated a lot of excitement within the cancer research field. Despite many approvals for various cancers, immunotherapeutic approaches are yet to be approved for the treatment of PDAC. Targeting KRAS G12D using engineered T cells is an exciting development for immunotherapy in the PDAC KRAS G12D space.

To answer the question, has the KRAS G12D fortress been conquered? At this point in time, there are definitely exciting advances towards that goal, but G12D agents have a few more hurdles to overcome. The fortress may not have been conquered yet, but the walls we once thought were impervious have certainly been breached.

## Author contributions

SB researched and drafted the article. HK and AA supervised the content. All authors contributed to the article and approved the submitted version.

## Funding

Work in the lab of AA is supported by NIH NCI R01CA24060701 and R37CA215427

## Acknowledgments

AA acknowledges support from SKY Foundation Inc and U CAN-CER VIVE foundation. SB is supported by WSU Thomas C. Rumble Fellowship.

## Conflict of interest

The remaining authors declare that the research was conducted in the absence of any commercial or financial relationships that could be construed as a potential conflict of interest.

AA received funding from Karyopharm Therapeutics Inc and Purple Biotech. AA serves as a consultant for GLG and Guidepoint.

## Publisher’s note

All claims expressed in this article are solely those of the authors and do not necessarily represent those of their affiliated organizations, or those of the publisher, the editors and the reviewers. Any product that may be evaluated in this article, or claim that may be made by its manufacturer, is not guaranteed or endorsed by the publisher.
